# Factors Associated with Screening Mammogram Uptake among Women Attending an Urban University Primary Care Clinic in Malaysia

**DOI:** 10.3390/ijerph19106103

**Published:** 2022-05-17

**Authors:** Nasturah Abdullah, Noorhida Baharudin, Mariam Mohamad, Mohamed-Syarif Mohamed-Yassin

**Affiliations:** 1 Department of Primary Care Medicine, Faculty of Medicine, Universiti Teknologi MARA, Selayang Campus, Batu Caves 68100, Selangor, Malaysia; nasturahabdullah@gmail.com (N.A.); syarif8258@uitm.edu.my (M.-S.M.-Y.); 2 Department of Public Health Medicine, Faculty of Medicine, Universiti Teknologi MARA, Sungai Buloh Campus, Jalan Hospital, Sungai Buloh 47000, Selangor, Malaysia; mariammd@uitm.edu.my

**Keywords:** breast cancer screening, health belief, mammogram, primary care, Malaysia

## Abstract

Screening mammograms have resulted in a reduction in breast cancer mortality, yet the uptake in Malaysia was low. This study aimed to determine the prevalence and factors associated with screening mammogram uptake among women attending a Malaysian primary care clinic. A cross-sectional study was conducted among 200 women aged 40 to 74 attending the clinic. The data was collected using questionnaires assessing sociodemographic, clinical characteristics, knowledge and health beliefs. Multiple logistic regression was used to identify factors associated with mammogram uptake. The prevalence of screening mammograms was 46.0%. About 45.5% of women with high breast cancer risk had never undergone a mammogram. Older participants, aged 50 to 74 (OR = 2.57, 95% CI: 1.05, 6.29, *p*-value = 0.039) and those who received a physician’s recommendation (OR = 7.61, 95% CI: 3.81, 15.20, *p*-value < 0.001) were more likely to undergo screening mammography. Significant health beliefs associated with mammogram uptake were perceived barriers (OR = 0.81, 95% CI: 0.67, 0.97, *p*-value = 0.019) and cues to action (OR = 1.30, 95% CI: 1.06, 1.59, *p*-value = 0.012). Approximately half of the participants and those in the high-risk group had never undergone a mammogram. Older age, physician recommendation, perceived barriers and cues to action were significantly associated with mammogram uptake. Physicians need to play an active role in promoting breast cancer screening and addressing the barriers.

## 1. Introduction

Breast cancer is the commonest cancer among women in most parts of the world, including Malaysia [1,2]. Based on the Malaysian National Cancer Registry Report, breast cancer accounted for 33.9% of all cancers among females in Malaysia, with a peak age of 60 to 64 years. The age-standardized (world standard population) incidence rate for breast cancer was 34.1 cases per 100,000 women (years 2012 to 2016). This had increased compared to the previous years (years 2007 to 2011), where an age-adjusted rate of 31.1 cases per 100,000 women was reported. The report also stated that the percentage of late diagnoses (locally advanced breast cancer and metastatic breast cancer) had increased from 43.2% (years 2007 to 2011) to 47.9% (years 2012 to 2016) [2]. Early detection of breast cancer leads to an almost complete cure, and with timely diagnosis and effective treatment, the 5-year survival rate can increase to 90 percent [3].

Mammography had been established as the standard screening method for breast cancer [4,5]. Meta-analyses of 11 randomized control trials showed a 20.0% reduction in breast cancer mortality rate among women who had mammography screening [6]. According to the Malaysian Clinical Practice Guidelines (CPG) on Breast Cancer 2019, screening mammograms should be offered to eligible women based on their age and risk category. High-risk women include those with BReast CAncer (BRCA) gene mutation carriers, first-degree relatives of BRCA mutation carriers, a history of chest irradiation at a young age, and first and/or second-degree family history of breast and/or ovarian cancer. Women who did not report any of these criteria were classified as average-risk. Biennial mammography was recommended for average-risk women aged 50 to 74. For high-risk women, screening was recommended annually between the ages of 40 to 50, and biennially thereafter [5].

In health education, multiple theoretical frameworks had been used to study health behaviors, including the Health Belief Model (HBM), which was developed by social psychologists from the United States in the 1950s [7]. The domains in the HBM include perceived susceptibility, perceived severity, perceived benefits, perceived barriers, self-efficacy, cues to action and health motivation. According to the HBM, a person’s health behavior depends on the belief about the impact of the illness and its consequences, provided that the person has a distinct course of action by which to proceed [7]. Based on this model, various questionnaires had been developed to assess health beliefs and behaviors, including questionnaires on breast cancer screening uptake [8,9,10]. In Malaysia, the HBM-based questionnaire was developed following a robust methodology in 2019. It was designed to assess health beliefs related to breast self-examination (BSE) and screening mammography among Malaysian women. It is available in both English and Malay languages [11].

Many factors were found to be associated with screening mammogram uptake. These include personal characteristics (sociodemographic and clinical factors), knowledge, awareness and attitude of breast cancer [12,13]. Previous studies had shown mixed findings in terms of the association between knowledge of breast cancer and screening mammogram uptake. Some studies showed a positive association [14,15], while others did not demonstrate significant findings [16,17]. In addition, physician adherence to screening mammogram guidelines, and cultural and health beliefs regarding breast cancer were also found to be significant factors associated with mammogram uptake [12,18,19]. Over the years, mixed findings regarding the association of health beliefs and breast cancer screening uptake were discovered internationally, as well as in Malaysia. Some studies showed that perceived susceptibility to breast cancer was significantly associated with screening mammogram uptake among women [20,21], while other studies showed that screening mammograms were not related to individual health beliefs [16,19]. Studying the factors associated with screening mammogram uptake is useful to allow targeted and specific interventions, which will hopefully improve the breast cancer screening uptake and the subsequent reduction in breast cancer related mortality.

In Malaysia, opportunistic breast cancer screening through mammography is offered to eligible women based on their age and risk category [5,22]. However, only 25.0% of Malaysian women aged 40 years and above were reported to have undergone a screening mammogram [23]. Furthermore, the prevalence of screening mammograms according to breast cancer risk category has not been reported. Thus, this study aimed to determine the prevalence of screening mammogram uptake, and its association with the sociodemographic and clinical characteristics, as well as the knowledge and health beliefs on breast cancer and mammogram, of patients attending an urban university primary care clinic in Malaysia.

## 2. Materials and Methods

### 2.1. Study Design and Population

This was a cross-sectional study conducted among asymptomatic women attending an urban university primary care clinic in Selangor, Malaysia. The majority of patients who attended this clinic were residents from the surrounding Gombak district, as well as the students, staff and the former staff of the university. The clinic offered walk-in services for acute care, long-term follow-up care and chronic diseases management, as well as preventative care.

The inclusion criteria for this study were asymptomatic women aged 40 to 74 years who attended this clinic during the study period and were able to read and write in Malay or English. For this study, asymptomatic women were defined as those who did not have any active complaint of breast abnormality. Meanwhile, the exclusion criteria were women with a current or previous history of breast cancer or benign breast condition, such as fibroadenoma or fibrocystic breast disease. Women who had reported psychiatric illnesses which may impair their ability to answer the questionnaires reliably were also excluded.

### 2.2. Study Tool

This study was conducted using a set of self-administered questionnaires, which consisted of sociodemographic characteristics, clinical information, knowledge and health beliefs on breast cancer and screening mammogram. The knowledge and health beliefs components were assessed using the Malaysian HBM Questionnaire© [11].

The Malaysian HBM Questionnaire© was developed as an instrument to assess health beliefs toward BSE and screening mammograms among Malaysian women [11]. This self-administered questionnaire was developed with a robust methodology based on the literature review of the existing instruments and health beliefs theory [8,9,10,11]. The first part of this questionnaire consists of 32 questions on the knowledge of breast cancer and mammogram, which had “Yes” and “No” response options. There were seven questions on symptoms of breast cancer, seven questions on risk factors of breast cancer, seven questions on method of breast cancer screening, two questions on perception of the breast lumps and nine questions on knowledge of mammography. One point is given for a correct answer and no points are given for incorrect answers. The total score ranges from 0 to 32 points, with higher points indicating better knowledge regarding breast cancer.

The second part of this questionnaire covered health beliefs about breast cancer and screening mammogram. This section was adopted from the Malaysian HBM Questionnaire©. The original HBM questionnaire consisted of 54 items framed within nine domains. Two domains specifically assessed health beliefs towards BSE, while seven domains assessed health beliefs towards breast cancer and screening mammogram. Based on the recommendation by the Malaysian CPG on Breast Cancer 2019 [5], BSE was no longer recommended as a screening method for breast cancer. Following consultation and approval by the developer of the HBM questionnaire, only the seven domains pertaining to health beliefs towards breast cancer and screening mammogram were used in this study. This was appropriate, as each domain can be used on its own and was scored independently [11]. The 38 items pertaining to breast cancer and screening mammogram were framed within seven domains which were: perceived susceptibility of breast cancer (five items), perceived severity of breast cancer (four items), health motivation (five items), perceived benefits of mammogram (five items), self-efficacy of mammogram (eight items), perceived barriers of mammogram (four items) and cues to action for the mammogram (seven items). The questionnaire was scored using a Likert scale ranging from one (strongly disagree) to ten (strongly agree). The total score for each domain was divided by the number of items in each domain. All the items in this questionnaire were positively worded except for items in the perceived barriers domain. The final score for each domain ranged from 1 to 10. The higher the score in each domain indicates that a woman is more likely to adopt screening behavior except for the perceived barriers domain, where higher scores indicate a lower likelihood of adopting screening behavior. The Cronbach’s alpha coefficient of the domains for breast cancer and screening mammogram ranged from 0.829 to 0.989, indicating good reliability [11].

### 2.3. Variable Definition

The dependent variable of this study was the screening mammogram uptake. The participants were considered to have undergone screening if they had a mammogram at least once in their lifetime.

The independent variables include sociodemographic and clinical characteristics, as well as knowledge and health beliefs. For sociodemographic characteristics, education level was grouped into primary, secondary and tertiary. Primary education was defined as formal schooling from 7 to 12 years old, while secondary education was schooling between 13 to 17 years old. Tertiary education was defined as an attainment of college or university education.

With regards to household income, participants were divided into three categories according to the classification by the Department of Statistics, Malaysia (DOSM). These were the bottom 40% (B40), middle 40% (M40) and top 20% (T20) [24]. The B40 was defined as a monthly household income of less than MYR4850, M40 was between MYR4850 and MYR10959, and T20 was above MYR10959.

As for clinical characteristics, participants’ body mass index (BMI) was classified as: low BMI (<18.5 kg/m^2^), normal BMI (18.5–22.9 kg/m^2^), overweight (23–27.4 kg/m^2^) and obese (>27.4 kg/m^2^) [25].

Breast cancer risks were categorized according to the Malaysian CPG on Breast Cancer 2019 [5]. The high-risk group was defined as self-reported first and/or second-degree family history of breast and/or ovarian cancer, and/or personal history of chest irradiation treatment. Chest irradiation was defined as any radiotherapy treatment to the chest. The participants who did not report any of these criteria were classified as average-risk [5].

### 2.4. Sample Size Determination

The sample size was calculated based on a study conducted by Yusof et al. [16]. Among women attending a primary care clinic in Selangor, 13.2% of them had undergone a screening mammogram [16]. Based on this, using a single proportion formula, taking the α value of 0.05 with an absolute precision of 5.0%, the minimum calculated sample size was 177.

### 2.5. Sampling Method, Participant Recruitment and Data Collection Procedure

The recruitment period was from December 2020 until June 2021. The non-probability convenience sampling method was used to recruit participants. Women who attended the university primary care clinic during the recruitment period were screened for eligibility according to the inclusion and exclusion criteria. Eligible patients were then invited to participate. Next, those who were interested to participate were given the information sheet regarding the study, which included the background, purpose, benefits, study procedures and contact details of the researchers. Written informed consent was obtained.

**Figure 1:**
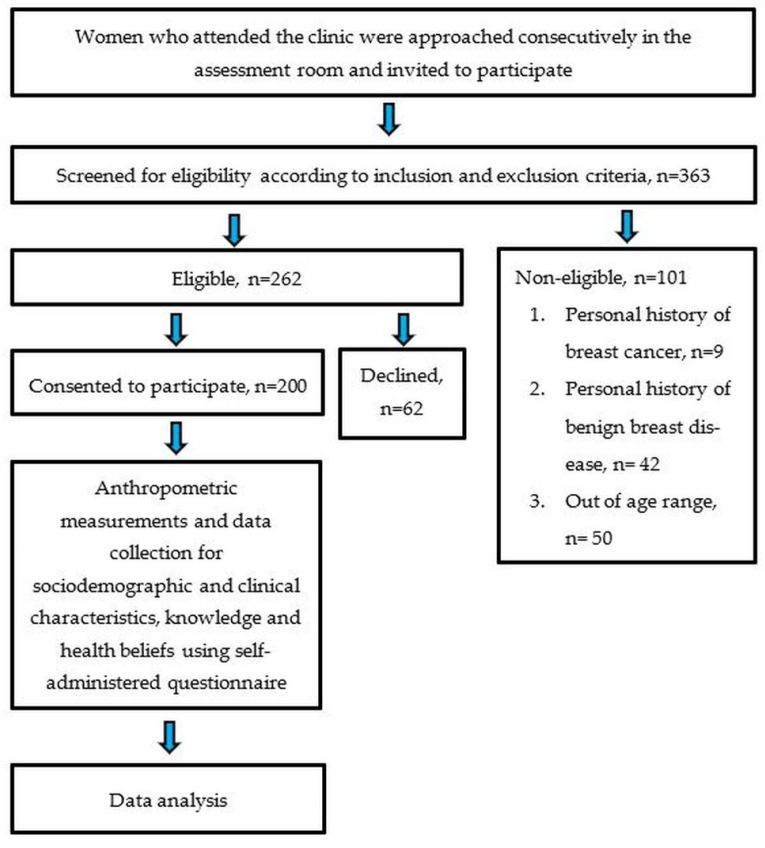
Flow chart of the study.

The questionnaires were given to the participants to be self-administered. Clear verbal instructions were given on how to complete the form. The participants were free to seek clarification from the researcher at any time if any enquiries arose. The filled questionnaires were returned directly to the researcher. These were then checked for completeness before the participants left the clinic. The conduct of the study is outlined in Figure 1.

The anthropometry measurements, i.e., weight and height, were performed by the researcher in the assessment room. Weight in kilograms (kg) and height in meters (m) were measured using the adult weighing machine and stadiometer (seca 787, seca, Hamburg, Germany). Weight was measured to the nearest 0.1 kg when the participant was standing on the scale with light clothing and without footwear. Height was measured to the nearest 0.01 m when the participant was standing on the same scale facing forward with the back, buttocks and heels against the scale. Two readings for each weight and height were measured and the average measurements were recorded. The participants’ body mass index (BMI) was calculated using the formula: BMI = weight (kg)/[height (m)]^2^ [25].

### 2.6. Data Entry and Statistical Analysis

Data entry and statistical analysis were performed using the latest IBM^®^ Statistical Package for Social Sciences (SPSS) version 27 program (IBM Corp., Armonk, NY, USA) [26]. Descriptive statistics were used to identify the respondent’s sociodemographic and clinical characteristics. Mean and standard deviations (SD) were used to describe continuous variables, while frequencies and percentages were used for categorical data. The Chi-square test was used to test the differences in proportion. Inferential analysis was conducted to identify factors associated with screening mammogram uptake. Simple logistic regressions were used to estimate the crude odds ratio (OR) of the factors associated with screening mammogram uptake. The variables with a *p*-value of < 0.05 were subsequently included in the multiple logistic regression (MLR) model using the forward LR method, to adjust for confounders. The factors associated with screening mammogram uptake were expressed as adjusted OR. Model fitness was checked using the Hosmer–Lemeshow goodness-of-fit test. Interactions, multicollinearity and assumptions were also checked. Statistical significance was taken at a *p*-value of < 0.05.

## 3. Results

### 3.1. Sociodemographic and Clinical Characteristics of the Participants

A total of 262 eligible women were invited to participate in the study. Sixty-two of them declined to participate. Therefore, the total number of participants was 200. The response rate was 76.3%. The mean (SD) age of the participants was 56.03 (7.75). The majority of the participants were in the 50 to 74 age group (79.0%), Malay (88.5%) and married (84.5%). Most of the participants had tertiary education (58.0%) and were pensioners/unemployed (58.0%). In terms of clinical characteristics, 17.0% of the participants had first and/or second-degree family history of breast and/or ovarian cancer, while 16.5% had a high risk for breast cancer. The participant’s sociodemographic and clinical characteristics are shown in Table 1.

### 3.2. Screening Mammogram Uptake

About 46% of the participants reported to have undergone a screening mammogram at least once in their lifetime. The majority of them (76.1%) had undergone the screening more than 2 years ago. Among the participants aged between 50 and 74 years old, about 51.3% had undergone a screening mammogram. More than half (54.5%) of those in the high breast cancer risk group had a mammogram performed. Table 2 shows the prevalence of screening mammogram uptake among the participants.

### 3.3. Knowledge and Health Beliefs on Breast Cancer and Mammogram

In this study, the mean (SD) knowledge score was 16.37 (6.30). The health belief score was lowest in the perceived susceptibility domain with a mean (SD) score of 4.34 (2.23) and highest in the cues to action domain with a mean (SD) score of 8.07 (1.73). Table 3 demonstrates the scores on knowledge and each domain of the health beliefs of the participants.

### 3.4. Factors Associated with Screening Mammogram Uptake

Table 4 shows the result from the simple logistic regression (SLR). Nine variables were found to have a *p*-value of < 0.05. These were age group, awareness of breast cancer screening program, physician recommendation for mammogram, knowledge score and five domains of the health beliefs (health motivation for breast cancer screening, perceived benefit of mammogram, perceived barriers for a mammogram, self-efficacy for mammogram and cues to action for the mammogram). These factors were included in the multiple logistic regression (MLR).

Table 5 shows the factors associated with screening mammogram uptake using multiple logistic regression analysis. The model fitness was assessed using Hosmer–Lemeshow goodness-of-fit test, which was not significant (*p* = 0.27). These indicated that the model fits the data well. The model explained 36.8% (Nagelkerke R Square) of the variance in the uptake of screening mammograms. The classification table demonstrated a specificity of 80.6%, indicating that the model could correctly classify 80.6% of the participants who did not attend screening mammography. The model’s sensitivity was 72.8%, indicating that the model could accurately classify 72.8% of participants who attended screening mammography. This model was able to discriminate 81.3% (ROC = 0.813, 95% CI: 0.75, 0.87, *p* < 0.05) of participants who attended screening mammography or not.

Based on the multiple logistic regression analysis, four factors were significantly associated with screening mammogram uptake. Older women (50 to 74) were 2.6 times more likely to undergo screening mammogram compared to younger women (40 to 49) (OR = 2.57, 95% CI: 1.05, 6.29, *p*-value = 0.039). Women who had ever received a physician recommendation for a mammogram had almost eight times the odds of undergoing mammogram screening (OR = 7.61, 95% CI: 3.81, 15.20, *p*-value < 0.001), compared to those who never received such recommendation. Women with a higher perceived barrier score were less likely to undergo screening mammogram (OR = 0.81, 95% CI: 0.67, 0.97, *p*-value = 0.019). Lastly, women who had a higher score in the cues to action domain had approximately 1.3 times the odds of undertaking a screening mammogram while controlling for other factors (OR = 1.30, 95% CI: 1.06, 1.59, *p*-value = 0.012).

## 4. Discussion

The prevalence of screening mammogram uptake among our study population was 46.0%. This was higher compared to the report produced by the National Institute of Health (NIH) Malaysia, where the national prevalence of screening mammograms was 25.0%. This report also found a lower prevalence of screening mammography uptake in Selangor (26.0%) [23]. A similar pattern was also seen in comparison with Yusof et al., who conducted their study in a primary care setting where the prevalence of screening mammograms in their study population was much lower (13.2%), compared to us. Our clinic consisted of family medicine specialists and doctors undergoing postgraduate training in primary care. Previous studies reported greater knowledge, awareness, confidence and practice in various areas among primary care doctors with a postgraduate qualification in Malaysia compared to those without [27,28]. The physicians in our clinic may potentially have greater knowledge and awareness of guideline recommendations of breast cancer screening, thus they would be able to recommend appropriate screening mammograms for their patients. This may explain the higher prevalence of screening mammogram uptake in our study. These findings highlighted the need to ensure that all primary care doctors received a postgraduate qualification in order to improve health outcomes for patients. Compared with developed countries, the studies conducted in the United States and England showed higher screening mammogram uptake, ranging from 66.7% to 69.1% [29,30]. This could be contributed by the nationwide population-based screening that they had adopted [30,31]. In Malaysia, nationwide population-based screening is yet to be implemented, and screening is normally done opportunistically when patients attend the clinics for other complaints [22]. For this reason, the practice related to breast cancer screening varied, with some states demonstrating higher screening rates than others, ranging from 7.0% to 38.9% [23].

In our study, we found that half (51.3%) of the participants from the older age group (50 to 74) had undergone a screening mammogram at least once in their lifetime. Various guidelines, including the Malaysian CPG on Breast Cancer 2019, had recommended screening mammograms for women in this age group [4,5]. Although the Malaysian guidelines recommended biennial screening mammograms for women in this age group, a majority (76.1%) of our participants had a screening mammogram more than two years ago. This may be explained by the pandemic, which had resulted in a lower clinic and hospital visits compared to the pre-Coronavirus Disease (COVID-19) time [32,33,34]. Other studies also discovered that patients were less likely to return for a screening mammogram after health facilities re-open following COVID-19-related closure [35,36]. While our study discovered that women aged 50 to 74 years old were twice more likely to undergo screening mammograms compared to the younger age group (40 to 49), the prevalence of screening mammogram uptake among them was still suboptimal. Thus, more rigorous interventions are needed to improve the uptake of breast cancer screening in this group of patients.

Various tools had been developed to assess breast cancer risk among patients, such as the Breast and Ovarian Analysis of Disease Incidence and Carrier Estimation Algorithm (BOADICEA) and the Gail model [37,38]. These algorithms, however, are not suitable for use among the Asian population as it tends to overestimate breast cancer risks [5,39]. Thus, the Malaysian CPG on Breast Cancer 2019 classifies women into average and high-risk categories [5]. In this study, the proportion of participants who attended screening mammography was higher in the high-risk group (54.5%) than in the average-risk group (44.3%), although this was not statistically significant. A study done in Germany showed a higher rate of regular mammogram screening (42.7%) in participants with a positive family history of breast cancer [40]. Earlier and regular screening mammograms in high-risk groups were recommended by the Malaysian guideline as it will lead to an earlier stage of diagnosis, better treatment options and a lower mortality rate [5]. Thus, clinicians should actively identify women at high risk of developing breast cancer by asking appropriate questions, such as a family history of breast and/or ovarian cancer, and recommend appropriate screening mammograms to achieve the best possible outcomes for this group of women [5].

In terms of the factors associated with screening mammogram uptake, this study found that physician recommendation was significantly associated with increased screening mammogram uptake. This finding was similar to a study conducted in the United States, which found that the participants who had ever received a physician’s recommendation for a screening mammogram were more likely to have it done [41]. Another study in a Kuala Lumpur tertiary hospital also found that physician recommendations would positively influence screening mammogram practice among its medical personnel [19]. Furthermore, the lack of physician recommendations was shown to lead to a lower screening rate among patients [42]. All these consistent findings suggest that physicians play an important role to promote cancer screening, specifically breast cancer screening among patients. The benefits of early detection and treatment of breast cancer had been clearly demonstrated [3], thus preventative care needs to be adopted actively by all physicians.

The cues to action refer to the stimulus which triggers the decision-making process to accept the suggested health action [43]. The cues include advice from the physicians and a health awareness campaign [43]. This study found a positive association between cues to action and screening mammogram uptake. Previous literature reported that a cue-to-action pilot project which consisted of outreach, education and an incentives program had resulted in an increased mammography screening rate by 170% [44]. In Malaysia, various breast cancer prevention measures have been developed and implemented, such as breast cancer awareness and mass media campaigns [45,46]. To complement these efforts, health promotion posters and breast cancer awareness campaigns should also be conducted locally at primary care clinics to trigger screening mammogram practice among women attending these clinics.

This study also discovered that perceived barrier was significantly associated with mammography practice. Perceived barrier refers to a person’s belief in barriers preventing them from performing health actions, such as undergoing a screening mammogram [43]. Women are more likely to participate in a screening procedure if they perceive the factors preventing them from taking the actions are less costly than its benefits [43]. Similar to our findings, a study done among Iranian women found that perceived barrier was inversely associated with screening mammogram practice [47]. Kirag and Kizilkaya’s study also shared these findings, in which perceived barriers were a significant negative predictor for screening mammogram practice [48]. A Malaysian study reported that lack of time, lack of knowledge and fear of the results were the most important barriers to breast cancer screening [49]. Shirzadi et al. explored such barriers in their qualitative study and found various individual, interpersonal, health system and community barriers which influenced women’s decisions to attend a breast cancer screening [50]. These findings provide insight for interventions to overcome the barriers, which will hopefully improve the rate of breast cancer screening among women.

Our study discovered that knowledge of breast cancer and mammogram was not a significant factor for breast cancer screening uptake. Previous Malaysian and international studies found mixed findings. Some studies showed a positive association between knowledge and screening mammogram uptake [14,15]. However, similar to our study, Yusof et al. and Kim et al. found that knowledge was not a significant predictor for screening mammogram uptake [16,17]. Although 58.0% of our study participants attained tertiary education, their mean knowledge score was only 16.37 out of 32 marks. The poor knowledge may have led to participants being unaware of their own risk factors for breast cancer.

### 4.1. Strengths, Limitations and Implications for Future Research

To the best of our knowledge, our study was the first to report on the prevalence of screening mammogram uptake according to breast cancer risk category. Although the results were not statistically significant, it has important clinical implications. Thus, it deserves to be studied further in future research.

This study has some limitations. First, this was a cross-sectional study that would only establish the association between the predictive variables and the screening mammogram practice. This study design would only show the relationship between variables, but not causality, thus the results should be interpreted in this context.

The convenience sampling method was used for participant recruitment, which may introduce selection bias. The women who attended the clinic on the data collection days, however, were approached consecutively in the nurse’s assessment room to minimize this bias.

This study was conducted from a single institution that consisted of Malays as the majority ethnic group. Other ethnic groups were underrepresented in this study. As a result, the findings from this study may not be generalizable to the general population with diverse ethnic groups, but they may be applicable to the clinics with the same sociodemographic distribution as our study. Thus, future studies should include participants from other primary care clinics to enable the generalization of the findings.

In view of the time limitation to conduct this study, other factors which may affect screening mammogram rate were not included. These factors include physician factors, such as their knowledge and attitude on breast cancer and screening mammogram, as well as their belief and confidence in diagnosing and managing breast cancer. Thus, this study was unable to determine the association between these factors with screening mammogram uptake and should be explored in future research. Understanding these factors would provide insight into the planning of a continuous medical education program, tailored to the needs of these medical professionals.

### 4.2. Implications for Clinical Practice

Our study findings have several implications for clinical practice. We found that clinicians play an important role in promoting breast cancer screening among women attending primary care clinics. The clinicians’ ability to provide appropriate breast cancer screening may be influenced by their knowledge and adherence to screening mammogram guidelines. These could be delivered via a continuous medical education program which would hopefully improve clinicians’ abilities to recommend appropriate breast cancer screening according to clinical indications, as suggested by the guidelines. Besides that, the clinician should also identify the patient’s barriers to undergo screening procedures, and facilitate measures to overcome these barriers.

Furthermore, various cues and prompts to encourage patients to attend screening mammograms should also be implemented in the clinics, such as sending reminders for a mammogram through online messaging services or emails. These measures can help to increase the rate of screening mammography among patients.

## 5. Conclusions

In conclusion, approximately half of the participants and those in the high-risk group had never undergone a screening mammogram. Older age group, physician’s recommendation, cues to actions and perceived barriers were significantly associated with screening mammogram uptake among women attending this primary care clinic. Physicians need to play an active role in promoting breast cancer screening, especially for women in the high-risk group, and in addressing their barriers to breast cancer screening.

## Figures and Tables

**Table 1:** Sociodemographic and clinical characteristics of the participants, n = 200.

Sociodemographic and Clinical Characteristics	Mean (SD)	Frequency, n (%)
**Age (years)**	56.03 (7.75)	
**Age group (years)**		
40–49		42 (21.0)
50–74		158 (79.0)
**Race**		
Malay		177 (88.5)
Chinese		11 (5.5)
Indian		12 (6.0)
**Marital status**		
Single/divorced/widow		31 (15.5)
Married		169 (84.5)
**Education level**		
Primary/secondary		84 (42.0)
Tertiary		116 (58.0)
**Occupation**		
Pensioner/unemployed		116 (58.0)
Employed		84 (42.0)
**Monthly household income**		
Bottom 40% (B40); <MYR4850		115 (57.5)
Middle 40% (M40); MYR4850 to MYR10959		74 (37.0)
Top 20% (T20); >MYR10959		11 (5.5)
**Have health insurance**		
No		130 (65.0)
Yes		70 (35.0)
**Comorbidities**		
Hypertension		112 (56.0)
Dyslipidemia		90 (45.0)
Diabetes mellitus		68 (34.0)
Asthma		20 (10.0)
**Breast cancer risk factors**		
Have ever used oral contraceptive pills (OCP)		50 (25.0)
Family history of breast and/or ovarian cancer		34 (17.0)
Have ever used hormone replacement therapy (HRT)		20 (10.0)
Nulliparity		18 (9.0)
Alcohol consumption		6 (3.0)
Past chest irradiation treatment		2 (1.0)
**Body mass index (kg/m2)**		
Underweight/normal		32 (16.0)
Overweight/obese		168 (84.0)
**Age of menarche (years)**		
>12		181 (90.5)
≤12		19 (9.5)
**Age of menopause ^ (years)**		
<50		113 (80.7)
≥50		27 (19.3)
**Breast cancer risk category**		
Average-risk		167 (83.5)
High-risk		33 (16.5)
**Aware of breast cancer screening program**		
No		76 (38.0)
Yes		124 (62.0)
**Have ever performed breast self-examination (BSE)**		
No		34 (17.0)
Yes		166 (83.0)
**Have ever received physician recommendation for a mammogram**		
No		120 (60.0)
Yes		80 (40.0)
Paid for mammogram *		
No		62 (67.4)
Yes		30 (32.6)
Reason for mammogram *		
Personal interest		58 (63.0)
Physician recommendation		26 (28.3)
Others		8 (8.7)
Intend to undergo mammogram **		
No		45 (41.7)
Yes		63 (58.3)

^ Included only participants who had attained menopause, n = 140. * Included only participants who had undergone mammogram, n = 92. ** Included only participants who had never undergone mammogram, n = 108.

**Table 2:** Prevalence of screening mammogram uptake according to age and breast cancer risk category.

Variables	Yes	No	*p*-Value
**Have ever undergone mammogram, n (%)**	92 (46.0)	108 (54.0)	-
**Last mammogram done ^, n (%)**			
<1 year	8 (8.7)	-	-
1 to 2 years	14 (15.2)	-	-
>2 years	70 (76.1)	-	-
**Age (years), n (%)**			
40 to 49	11 (26.2)	31 (73.8)	0.004
50 to 74	81 (51.3)	77 (48.7)	
**Breast cancer risk category, n (%)**			
Average-risk	74 (44.3)	93 (55.7)	0.281
High-risk	18 (54.5)	15 (45.5)	

^ Included only participants who had undergone mammogram, n = 92.

**Table 3:** The mean scores for knowledge and health beliefs.

Variables	Mean (SD)	Minimum	Maximum
**Knowledge score (0 to 32)**	16.37 (6.30)	2.00	28.00
Symptoms of breast cancer (0 to 7)	4.09 (2.37)	0.00	7.00
Risk factors of breast cancer (0 to 7)	2.16 (1.29)	0.00	6.00
Methods of breast screening (0 to 7)	4.09 (1.96)	0.00	7.00
Perceptions on breast lump(s) (0 to 2)	0.74 (0.84)	0.00	2.00
Knowledge on mammography (0 to 9)	5.30 (2.19)	0.00	9.00
**Health belief regarding breast cancer (1–10)**			
Perceived severity (1–10)	6.61 (2.07)	1.00	10.00
Perceived susceptibility (1–10)	4.34 (2.23)	1.00	10.00
Health motivation (1–10)	7.78 (1.71)	1.40	10.00
**Health belief regarding mammogram (1–10)**			
Perceived benefit (1–10)	7.94 (1.81)	1.00	10.00
Perceived barriers (1–10)	4.51 (1.93)	1.00	10.00
Self-efficacy (1–10)	7.37 (1.91)	2.25	10.00
Cues to action (1–10)	8.07 (1.73)	2.00	10.00

**Table 4:** Association of sociodemographic, clinical factors, knowledge and health beliefs with screening mammogram uptake.

Variables	Beta	Wald (df)	OR (95% CI)	*p*-Value
**Age group (years)**				
40–49			1	
50–74	1.09	7.95 (1)	2.97 (1.39, 6.31)	**0.005**
**Race**				
Malay			1	
Chinese	0.38	0.36 (1)	1.46 (0.43, 4.94)	0.548
Indian	0.19	0.10 (1)	1.21 (0.38, 3.91)	0.747
**Marital status**				
Single/divorced/widow			1	
Married	0.20	0.24 (1)	1.22 (0.56, 2.64)	0.622
**Education level**				
Primary/secondary			1	
Tertiary	−0.03	0.01 (1)	0.97 (0.55, 1.71)	0.918
**Occupation**				
Pensioner/unemployed			1	
Employed	−0.47	2.62 (1)	0.63 (0.35, 1.11)	0.106
**Monthly household income**				
Bottom 40% (B40); <MYR4850			1	
Middle 40% (M40); MYR4850 to MYR10959	−0.15	0.25 (1)	0.86 (0.48, 1.55)	0.617
Top 20% (T20); >MYR10959	0.30	0.23 (1)	1.36 (0.39, 4.69)	0.631
**Have health insurance**				
No			1	
Yes	0.25	0.69 (1)	1.28 (0.72, 2.29)	0.405
**Comorbidities**				
Diabetes mellitus	−0.25	0.01 (1)	0.98 (0.54, 1.75)	0.933
Hypertension	−0.45	2.48 (1)	0.64 (0.36, 1.12)	0.115
Dyslipidemia	0.05	0.03 (1)	1.05 (0.60, 1.84)	0.864
Asthma	0.63	1.71 (1)	1.88 (0.73, 4.81)	0.191
**Breast cancer risk factors**				
Family history of breast and/or ovarian cancer	0.34	0.79 (1)	1.40 (0.67, 2.93)	0.374
Have ever used oral contraceptive pills (OCP)	0.00	0.00 (1)	1.00 (0.53, 1.90)	1.000
Have ever used hormone replacement therapy (HRT)	0.87	3.09 (1)	2.37 (0.91, 6.23)	0.079
Alcohol consumption	0.17	0.04 (1)	1.18 (0.23, 5.99)	0.842
Nulliparity	−0.07	0.02 (1)	0.93 (0.35, 2.47)	0.890
**Body mass index (kg/m^2^)**				
Underweight/normal			1	
Overweight/obese	−0.49	1.59 (1)	0.61 (0.29, 1.31)	0.207
**Age of menarche (years)**				
>12			1	
≤12	0.06	0.02 (1)	1.06 (0.41, 2.74)	0.900
**Age of menopause ^ (years)**				
<50			1	
≥50	0.02	0.00 (1)	1.02 (0.44, 2.37)	0.961
**Breast cancer risk category**				
Average-risk			1	
High-risk	0.41	1.15 (1)	1.51 (0.71, 3.19)	0.283
**Aware of breast cancer screening program**				
No			1	
Yes	0.78	6.75 (1)	2.19 (1.21, 3.95)	**0.009**
**Have ever performed breast self-examination (BSE)**				
No			1	
Yes	0.24	0.38 (1)	1.27 (0.60, 2.68)	0.536
**Have ever received physician recommendation for a mammogram**				
No			1	
Yes	2.11	40.75 (1)	8.25 (4.32, 15.77)	**<0.001**
**Knowledge score**	0.05	4.67 (1)	1.05 (1.01, 1.10)	**0.031**
**Health belief regarding breast cancer**				
Perceived severity	0.08	1.20 (1)	1.08 (0.94, 1.24)	0.274
Perceived susceptibility	0.06	0.80 (1)	1.06 (0.93, 1.20)	0.372
Health motivation	0.22	5.97 (1)	1.24 (1.04, 1.48)	**0.015**
**Health belief regarding mammogram**				
Perceived benefit	0.21	5.65 (1)	1.23 (1.04, 1.46)	**0.017**
Perceived barriers	−0.21	7.41 (1)	0.81 (0.70, 0.94)	**0.006**
Self-efficacy	0.21	7.42 (1)	1.24 (1.06, 1.45)	**0.006**
Cues to action	0.27	8.70 (1)	1.30 (1.09, 1.56)	**0.003**

1 = Reference group. Emboldened: Statistical significance at *p* < 0.05. ^ Included only participants who had attained menopause, n = 140.

**Table 5:** Factors associated with screening mammogram uptake.

Variables	Adjusted Beta	Wald (df)	Adjusted OR (95% CI)	*p*-Value
**Age group (years)**				
40–49			1	
50–74	0.94	4.26 (1)	2.57 (1.05, 6.29)	0.039
**Have ever received physician recommendation for a mammogram**				
No			1	
Yes	2.03	33.02 (1)	7.61 (3.81, 15.20)	<0.001
**Health belief regarding mammogram**				
Perceived barriers	−0.22	5.54 (1)	0.81 (0.67, 0.97)	0.019
Cues to action	0.26	6.26 (1)	1.30 (1.06, 1.59)	0.012

The model fits well (*X*^2^ = 64.51, df = 4, n = 200, *p* < 0.05). Model assumptions were met, no significant interaction and multicollinearity.

## Data Availability

Data are kept at the Department of Primary Care Medicine, Universiti Teknologi MARA, in Selangor, Malaysia. Data may be shared upon reasonable request and are subjected to data protection laws and regulations.
